# High-Dimensional Contact Network Epidemiology

**DOI:** 10.3390/epidemiologia4030029

**Published:** 2023-07-07

**Authors:** Andrew Ackerman, Briquelle Martin, Martin Tanisha, Kossi Edoh, John Paul Ward

**Affiliations:** 1 School of Mathematical and Statistical Sciences, Clemson University, Clemson, SC 29634, USA; atacker@g.clemson.edu; 2 Department of Mathematical Sciences, Appalachian State University, Boone, NC 28608, USA; martinb15@appstate.edu; 3 Department of Mathematics and Statistics, NC A&T State University, Greensboro, NC 27411, USA; tmthomas@aggies.ncat.edu (M.T.); jpward@ncat.edu (J.P.W.)

**Keywords:** epidemiology, graph theory, bond percolation

## Abstract

Contact network models are recent alternatives to equation-based models in epidemiology. In this paper, the spread of disease is modeled on contact networks using bond percolation. The weight of the edges in the contact graphs is determined as a function of several variables in which case the weight is the product of the probabilities of independent events involving each of the variables. In the first experiment, the weight of the edges is computed from a single variable involving the number of passengers on flights between two cities within the United States, and in the second experiment, the weight of the edges is computed as a function of several variables using data from 2012 Kenyan household contact networks. In addition, the paper explored the dynamics and adaptive nature of contact networks. The results from the contact network model outperform the equation-based model in estimating the spread of the 1918 Influenza virus.

## 1. Introduction

Network science research has become a productive research area since the 1990s. Related areas of research include the investigation of complex networks such as computer networks, biological networks, social networks, and contact networks in epidemiology. As computational power increases and numerical simulations improve, mathematical models for the spread of diseases have gone from simple equation-based models using mean field theory to models involving detailed information about the social interaction of individuals. In particular large-scale stochastic, individual-based simulations on contact networks have developed [1]. The network-based framework is more convenient for investigating different network connections, interactions, and evolutions with different initial conditions. Deriving system-level macro information to explain the epidemic dynamics from such detailed micro-level simulation seems a daunting task. The accuracy of network simulation models in predicting the spread of disease and the efficiency of intervention strategies depend on getting accurate representative static or dynamic contact networks of the population.

Among related work is the top-down approach using continuous Markov chains as well as the bottom-up approach from node level using nearest neighbors [2]. There has been the use of mean-field theory on homogeneous and heterogeneous networks, and percolation-based approach for disease modeling. The literature cites the use of network-based compartmental models in non-Markovian epidemic models as well as integrodifferential and delays equations, such as the Fokker–Plank equation, to model the distribution of the number of infected individuals as a function of time [2].

This paper investigates the computation of the weight of edges in contact networks as a function of several variables such as age, gender, node degree, number of contacts, and duration of contacts. Static contact networks are best used for modeling rapidly spreading pathogens such as Severe Acute Respiratory Syndrome (SARS) and pneumonia. For slow-spreading pathogens, adaptive and dynamic contact network models work better [3]. The results from our contact network models are more accurate in predicting the spread of the 1918 Influenza virus than the equation-based model. The rest of the paper is organized as follows: Section 2 contains the background theory, Section 3 the results, and Section 4 the discussions and conclusion.

## 2. Methods

The paper uses contact networks to model the spread of communicable diseases. Historically, this has been done through the use of systems of ordinary differential equations (ODEs) which rely on mass action assumption wherein each individual has an equally likely chance of being infected. Results from ODE models turn out to differ significantly from those observed in populations where the degree of the contacts deviate significantly from the mass action assumption.

Equation-based models are usually based on grouping the population in different compartments such as susceptible, asymptomatic/pre-symptomatic, infected, and recovered. Variants of this method include the Susceptible-Asymptomatic-Infected-Recovered-Susceptible (SAIRS) model, and the Susceptible-Exposed (Asymptomatic/pre-symptomatic)-Infected-Recovered model (SEIR). For comparison with our network models, we implemented SEIR known to be common to many diseases such as Influenza and COVID-19, where the disease may lay dormant within a host when exposed to the pathogen and not exhibiting outward symptoms of being infected. This period of a couple of days could be very influential in the overall spread of the disease.

### 2.1. SEIR Model

Our implementation of the equation-based model used the SEIR model. Let *S* denote the number of susceptible individuals, *E* the number of exposed individuals, *I* the number of infected individuals, and *R* the number of recovered individuals, such that S(t), E(t), I(t), and R(t) represent these respective quantities as functions of time. Where there are no births and deaths, the population size *N* is constant, and S(t)+E(t)+I(t)+R(t)=N.

Let λ represent the rate of recovery, ϵ the rate at which exposed individuals transition to an infected state, and β the rate at which an individual leaves the susceptible state and becomes exposed. Thus, a population can be modeled through a system of differential equations as follows [4]:

(1) $$\left.\begin{array}{cc} \frac{dS}{dt} & =\frac{-\beta IS}{N}\\ \frac{dE}{dt} & =\frac{\beta IS}{N}-\epsilon E\\ \frac{dI}{dt} & =\epsilon E-\lambda I\\ \frac{dR}{dt} & =\lambda I \end{array}\right..$$

The quantity 1/λ represents the average number of days it takes for an infected individual to recover [5].

### 2.2. Network Models

In network models, the nodes are individual entities (humans, animals, or cities) and the edges are the contacts between said entities. Among network models used in epidemiology are exact models, pairwise models, edge-based compartmental models, and bond percolation models. The overall susceptibility of a node depends on the degree of the node—the number of edges connected to the node. The transmission likelihood of an edge is reflected in the weight of the edge. Thus, the factors that determine the weight of an edge are of great interest to us. Among these factors are the following: the duration of contacts [6] and number of contacts between nodes on the edge; age and gender of nodes; pathogen strength; and geographic area [7].

In this research, the weight of an edge is the probability that a node if infected will transmit the disease to the other node on the edge. The weights can be symmetric or non-symmetric. Since the weight is dependent on several variables, we derive it as a function of these variables. The event of selecting each of these variables is independent of one another other. The weight is therefore the product of the probabilities of each of the variables. For example, the event of selecting an individual (node) with a specific gender is independent of the event of selecting an individual being in a specific age group. The probability of an edge is simply the product of the probabilities of the independent events. Equation-based models assume all susceptible individuals are equally likely to become infected whereas network models involve stochastic processes of interactions among individuals within a population. New members of the population are born or become deceased, members immigrate into or emigrate from an area, or simply alter with whom they associate. Within a network model, this can be reflected in the addition and deletion of nodes, and the rewiring of edges [8]. One particular type of dynamics utilized in this research is the tendency for humans to avoid individuals known to be infected [9].

The ultimate aim of many epidemiological studies is to propose, how the epidemic can be prevented or the spread of the disease can be reduced. Intervention methods include vaccination and social distancing, each of which has its strategic advantages. For instance, at the moment of exposure to a disease, social distancing may be the best intervention method [1]. However, social distancing may be of little to no effect if individuals identified as “super-spreaders”, have already been infected and potentially disseminated the disease. In this case, vaccination of the whole population may be more effective. If individuals on the fringes of the social network are the primary ones affected, it can be more effective and certainly more efficient to socially distance these individuals from the population.

#### Graph Theory

Graph theory provides us with mathematical theories and applications of graphs including visual displays of network data. Node centrality determines influential or central nodes in a network. The formal measures of node centrality are the degree, closeness, and betweenness of the node [10].

The degree of a focal node, in our case, is the number of nodes it is connected to and determines the ties that the focal node has. The closeness centrality of a node is the inverse sum of the shortest distances to all the other nodes. The closeness centrality $C_{c}\left(u\right)$ for a node *u* is defined as

$$C_{c}\left(u\right)=\frac{n-1}{\sum_{v\in V}d\left(u,v\right)},$$

where *n* is the number of nodes in the network, d(u,v) is the length of the shortest path from node *u* to node *v*, and *V* the set of nodes in the network. It determines how quickly the node can be reached from the other nodes. Betweenness centrality, by contrast, determines how a node controls the flow between other nodes and is determined by the number of times the node is traveled through when taking the shortest path from one node to another. The betweenness centrality $C_{B}\left(u\right)$ for a node *u* is defined as

$$C_{B}\left(u\right)=\sum_{s\neq u\neq t}\frac{\sigma_{st}\left(u\right)}{\sigma_{st}},$$

where $\sigma_{st}$ is the number of shortest paths with nodes *s* and *t* as their end nodes, whereas $\sigma_{st}\left(u\right)$ is the number of shortest paths that include node *u* [11].

**Figure 1:**
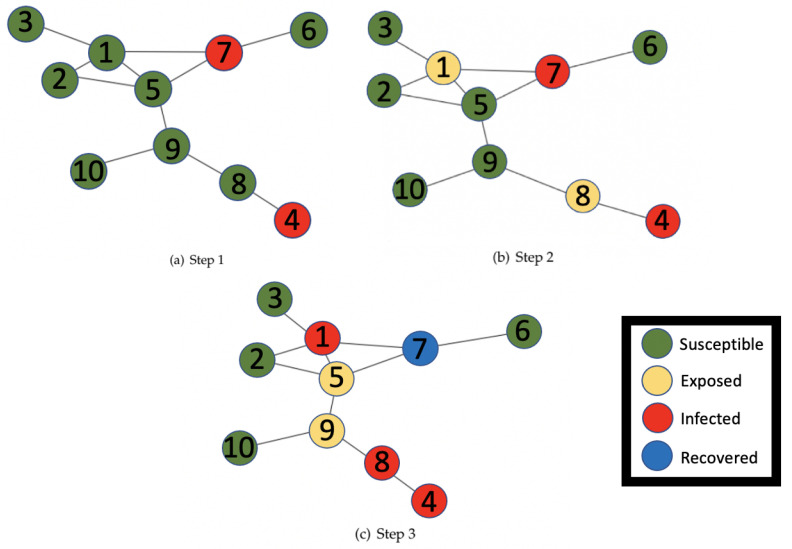
Network model of SEIR epidemic spread dynamics.

In Figure 1, we have provided an example of how an epidemic may spread through a contact network based on the SEIR model.

In step one of the network, nodes four and seven are infected leaving the rest of the nodes susceptible. In step two, the neighbors, nodes one and eight, of the infected nodes become exposed to the disease. Moving forward to step three, nodes one and eight now go from exposed to infected, whereas node seven goes from infected to recovered and in the process, nodes five and nine become exposed. For our model, we assume once a node is recovered, it will not become infected again. Typically, the edges in the graph can be directed or undirected depending on what they represent.

NetworkX is a Python package that can be used to create a variety of network structures. It can create graphs with a specified number of nodes and edges, networks of popular distributions such as Power-law and Watts–Strogatz, and compute parameters such as diameter, degree distribution, number of connected components, and node betweenness centrality [4]. For our purpose, we used NetworkX to create the images in Figure 1 to show the progress of the spread of the disease through our network.

### 2.3. Percolation

The dynamics of the spread of pathogens on contact networks are explained below using bond percolation theory. As shown in Figure 1, initial nodes are infected with the disease, and the disease begins to propagate through the network as described. The initial nodes remain infectious for a period of time, during which they have the potential to transmit the disease to their contacts. The secondary cases have the potential to transmit the disease to their contacts during their infectious period, and so on. This process is similar to bond percolation, a topic that has been extensively studied by mathematicians and physicists [12,13]. There are some similarities between epidemiology and bond percolation such as transmission probability and occupation probability, epidemic threshold and percolation threshold, probability and size of large-scale epidemic, and probability and size of the giant component.

In bond percolation each edge is said to be occupied with probability *T* and empty with probability 1−T where *T* is defined as the transmissibility: The overall probability that an infectious node on one end of the edge will transmit the pathogen across the given edge before it recovers. The percolation of an epidemic contact network depends on the weights of the edges and the structure of the network. An edge in the network is associated with a per unit time probability of disease transmission $r_{ij}$, that is, node *i*, if infected, will transmit the disease to node *j* within a given time. The probability $r_{ij}$ is dependent on several variables such as the number of contacts and duration of contacts between nodes *i* and *j*. Assuming discrete time steps, if node *i* is infectious for τ time steps, then the probability that *j* will be infected by *i* is $T_{ij}=1-{\left(1-r_{ij}\right)}^{\tau }$ and for continuous time intervals, $T_{ij}=1-e^{-r_{ij}\tau }$ [2]. Assume $r_{ij}$ to be an independent identically distributed random variable chosen from a distribution P(τ), then $T_{ij}$ is also an independent identically distributed random variable. The mean probability of transmission between individuals is given by

(2) $$T=1-\int_{0}^{\infty }1-P\left(\tau \right){\left(1-\tau \right)}^{\tau }\;d\tau ,$$

for discrete time [2].

### 2.4. Computation of Edge-Weights

The weight $r_{ij}$ is dependent on several variables such as pathogen strength, age and gender of individuals represented by the nodes, and the number of contacts per unit time. Let $x_{k}\left(t\right)$ denote the *k*th variable. Thus, $r_{ij}$ can be represented by

$$r_{ij}=f\left(\left\{x_{1}\left(t\right),x_{2}\left(t\right),x_{3}\left(t\right),\cdots ,x_{n}\left(t\right)\right\}\right),$$

where *n* is the number of variables. Let $\Psi_{k}^{ij}$ denote a probability of an event associated with the variable $x_{k}$. The events corresponding to the variables $x_{k},k=1,\cdots ,n$ are linearly independent; thus, the combined probability $r_{ij}$ is the product of all the probabilities of the independent variables.

(3) $$r_{ij}=\Psi_{1}^{ij}*\Psi_{2}^{ij}*\Psi_{3}^{ij}*...*\Psi_{n}^{ij}.$$

### 2.5. Dynamic Contact Network

In our model, we assumed that the contact networks are not static and that the weights and edges change with time. As individuals break or form friendships, new edges are removed or added to the contact network. A change in the number of contacts and the duration of contacts per unit time between two individuals results in a change in the weight of the corresponding edge. One such scenario is when a susceptible individual becomes infected there is a tendency for the other susceptible individuals to avoid contact with the infected individual reducing the weight of the edges associated with the infected node. This could be observed during the recent COVID-19 when patients self-isolated or quarantined alone. In the literature, it is observed that contact rates tend to increase with the density of the population, and the contact rates in crowds are estimated using the kinetic theory of the Van der Waals gas model [14].

In this project, we provide a simple model in which we let *c* represent the number of contacts per unit time that the infected node has with its neighbor before being infected. We assume that the number of contacts $\tilde{p}$ per unit time after the node is infected is proportional to *c*. Further, let α denote the proportionality constant such that $\tilde{p}=\alpha$c. The proportionality constant α, can be estimated from how fast people become aware that the individual is infected.

## 3. Results

### 3.1. Airline Data

The first experiment was conducted using airline data. This data contains airport locations in the United States and the number of passengers flying between airports during any given month of the year. When relating this to contact networks, the nodes are the airports and the number of passengers flying between airports in a given month are the weights of the edges. The weight in this case consists of only one variable, the number of passengers. To obtain the $r_{ij}$s, we divide the total number of passengers traveling either way between airports *i* and *j* by a value one (1) point higher than the largest value of the passengers traveling between the airports. For the results in our network model, we run the simulations one thousand (1000) times, each time initially infecting three randomly chosen nodes and then averaging the results. Running the simulation over 1000 times does not change the average. In our network model, we decrease the weight of the edges of a node by a constant factor of α=0.4 when it becomes infected. The value of the constant α may be determined based on the severity of the infection.

**Figure 2:**
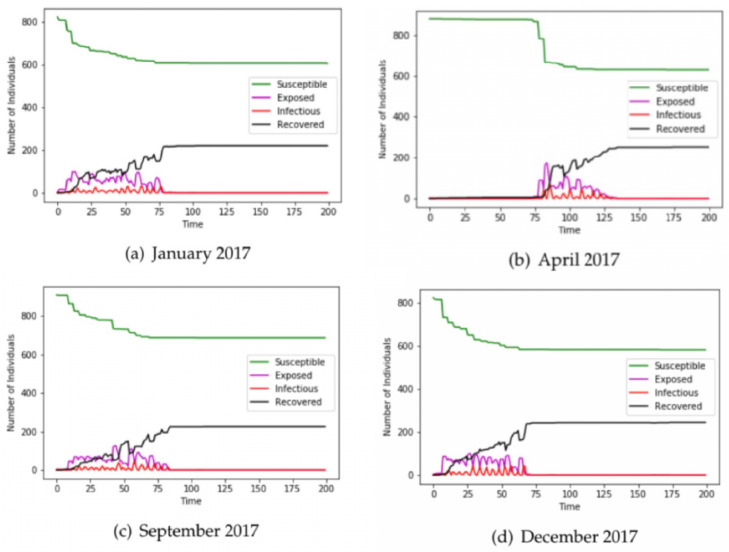
The number of susceptible, exposed, infected, and recovered nodes running the network model on 2017 Federal Aviation Administration flight data for 900 airports.

Figure 2a–d are the results of running the Federal Aviation Administration flight data sets for January, April, September, and December with the following parameters: β=0.411,ϵ=1/2.62,λ=1/3.38 for the 1918 Influenza virus [15]. For $R_{0}=\beta /\gamma =1.389$, the approximate final epidemic size $S_{\infty }=N\left(1-e^{-R_{0}}\right)=N\left(0.75\right)$, where *N* is the population size. The final size of the recovered nodes (*R*) is approximately 25% of the population. In our experiments, it will be shown that the final size of recovered nodes computed from the network model (30% of the population) is closer to the classical analytical result than that computed from the ODE model.

**Figure 3:**
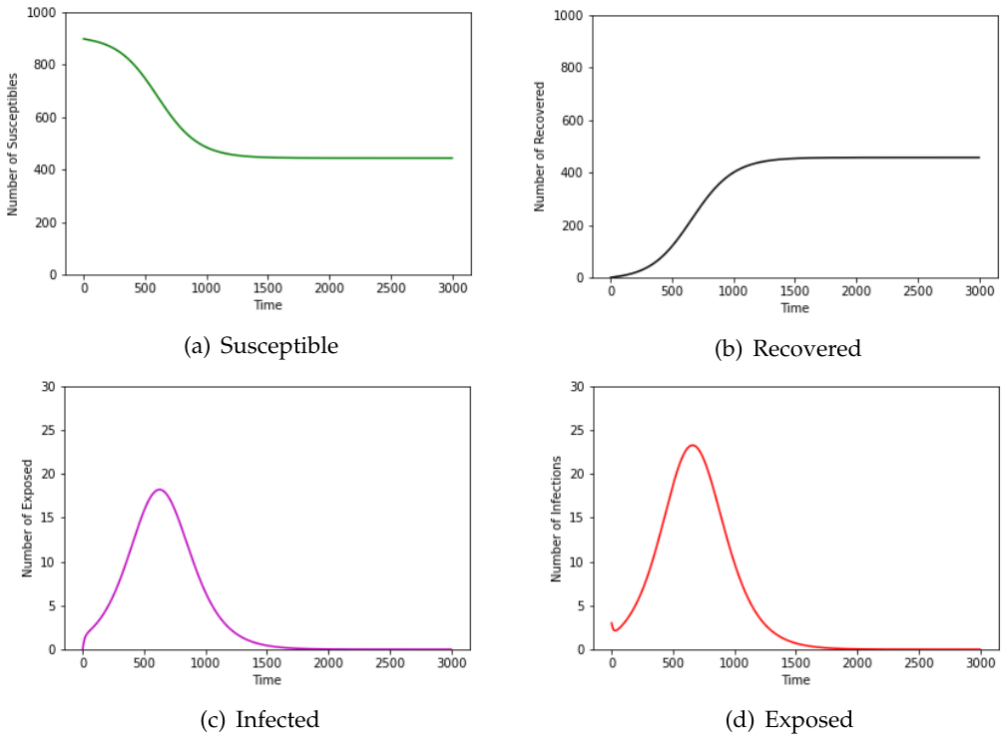
The number of susceptible, exposed, infected, and recovered nodes running the SEIR ODE model with the same parameter values as used in the network model for the airline data.

We chose airline data for these four months, as intuition would dictate that travel patterns deviate throughout different seasons of the year. As the disease is introduced in the population by infecting **three** susceptible airports among 900 chosen for this experiment, it is observed that the number of susceptible airports starts high and reduces to a lower value as susceptible airports become exposed and later infected. Further, the number of exposed and infected airports increased with time to some maximum and subsequently decline with the introduction of human intervention and countermeasures. Finally, the number of recovered airports increases from zero and grows inversely to the number of susceptible airports. Figure 3 contains the results of our ODE program with the same parameter values as used in the contact network model. According to Taubenberger [3], the bird flu (avian influenza) virus of 1918 infected around 33% of the world’s total population. The recovered airports from Figure 2 in the network model indicate that about 30% of the population was infected whereas Figure 3 from the ODE model shows that about 50% of the population was infected. In this experiment, the network model provided a more accurate estimate of the infected population than that of the ODE model.

### 3.2. Kenyan Household Data

**Figure 4:**
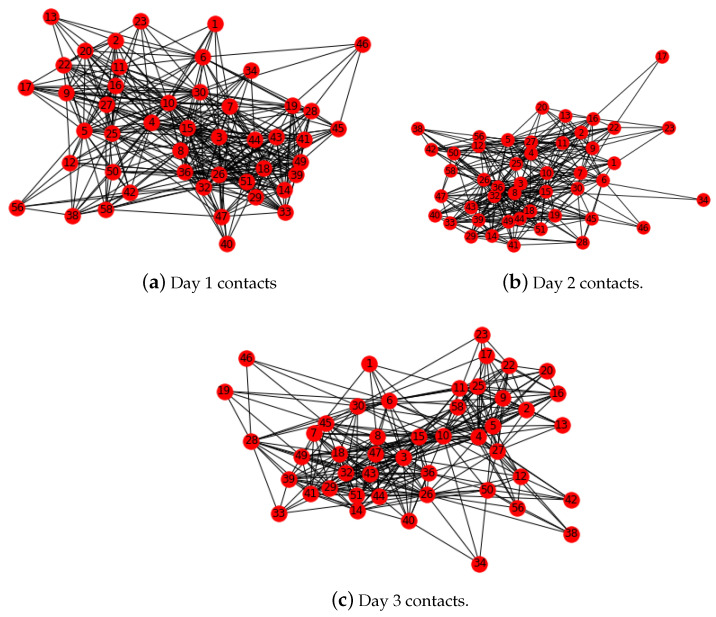
Kenyan household contacts.

The second data set was for Kenyan households. These data contain the contacts between members of five Kenyan households. These contacts occurred between 24 April and 12 May 2012. In Figure 4 are the Day 1, Day 2, and Day 3 networks for three chosen consecutive days. The data include member ID, age, gender, duration of contact, the day on which contact occurred, and the hour the contact occurred. We let the nodes in our network represent individuals in the different households, of which there are 60 in total. The edges and corresponding weights are determined by the duration of the contacts between the nodes on the edge, age, and gender of the nodes on the edge.

We used NetworkX to measure the degree distribution of the nodes and determine the most influential nodes in each daily network. We observed that the node with the highest degree 29 in Day 3 network had the highest degree 39 in the Day 2 network and the second highest degree 10 in the Day 1 network. Similar behavior is observed with degree centrality measure. The node with the highest centrality of 0.6304 in the Day 2 network has the highest centrality of 0.8478 in the Day 3 network and the second highest centrality 0.5735 in the Day 1 network. This would seem to suggest that this node is a highly influential member of the three networks. When infected, the sheer number of contacts would pose a considerable risk of transmission to the rest of the network. By contrast, if the node with the smallest centrality measure of 0.1304 is infected, the overall risk of transmission to the group as a whole would be far less.

**Figure 5:**
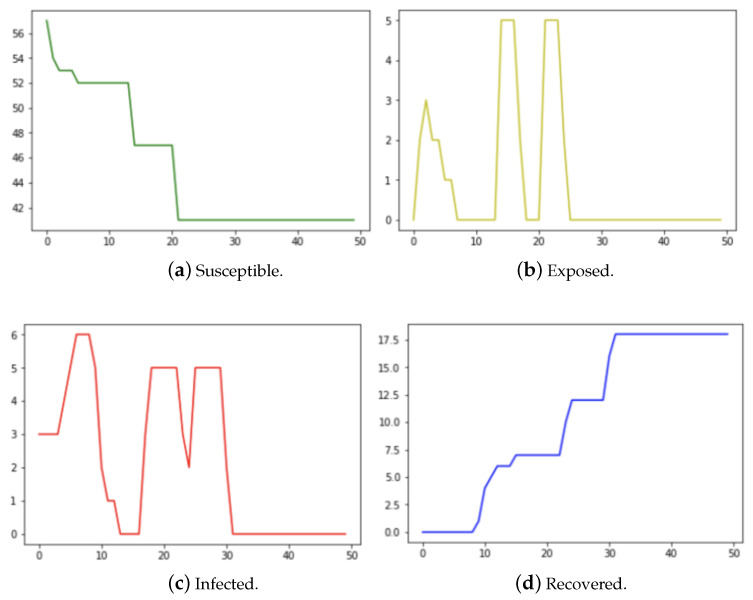
The number of susceptible, exposed, infected, and recovered nodes from the network model with edge-weights determined from a single variable—the contact duration in the Day 3 Kenyan household data.

**Figure 6:**
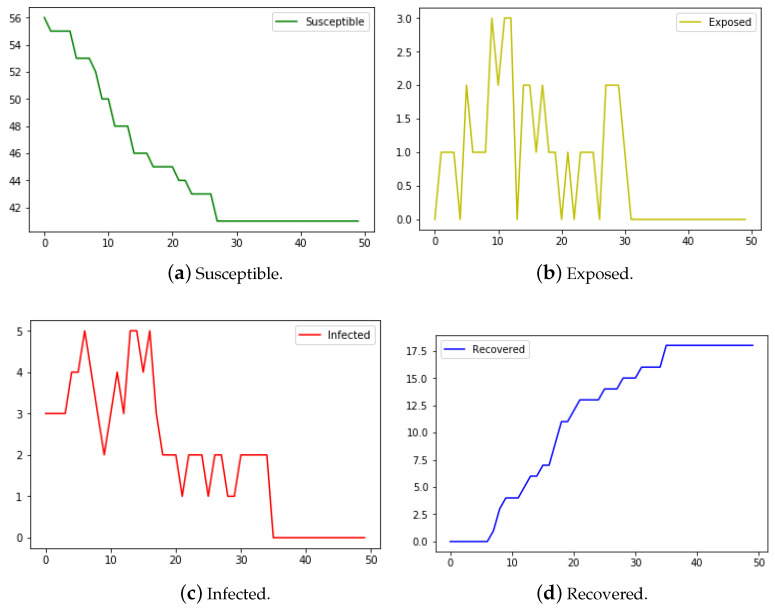
The number of susceptible, exposed, infected, and recovered nodes from the network model with the edge-weights determined from the contact duration, gender, and age in the Day 3 Kenyan household data.

**Figure 7:**
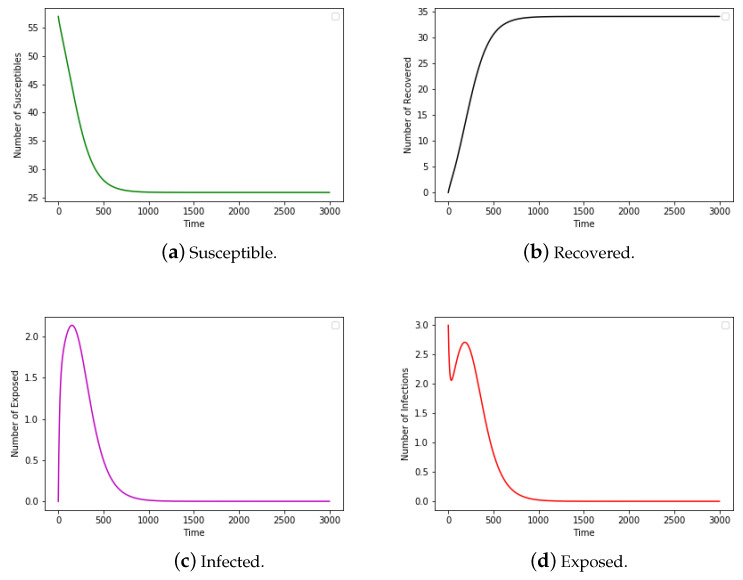
The number of susceptible, exposed, infected, and recovered nodes from the SEIR ODE model with the same parameter values as used in the network model for the Kenyan data.

The number of individuals in the networks in Figure 5, Figure 6 and Figure 7 is n=60. In Figure 5, the weight is dependent upon a single variable—total contact duration. The weight $r_{ij}$ is the total contact duration between individuals *i* and *j* divided by a value one point higher than the maximum value of total contact durations between individuals in the network.

We start with a susceptible sample size of n−3, as we initially infect three nodes. Out of 60 total individuals in the sample, 42 were left susceptible, indicating that 18 (30.0%) of the total were infected by the end of the transmission period. This is a marked improvement from the ODE model over the same sample which resulted in just about 27 individuals still susceptible by the end of the transmission, indicating that 33 (55%) were infected over the transmission period in Figure 7. It is worth noting that one of the main assumptions of the classic compartmental model approach requires that the population size be large. In this experiment, the population size of 60 may not be large enough that the fraction of individuals in each compartment can in good approximation be represented by a continuous value.

In Figure 6 we ran the same program using the Kenyan household data of 60 nodes. However, within this version, the weight of the nodes was determined from several variables—total contact duration, age, and gender. The graph of the contact network is undirected and the edge weights, $r_{ij}$s, are given by

(4) $$r_{ij}=\Psi_{1}^{ij}*\Psi_{2}^{ij}*\Psi_{3}^{ij},$$

where $\Psi_{1}^{ij}$ is the probability that the gender of individuals *i* and *j* corresponding to an elementary event in the sample space

{MaleMale,MaleFemale,FemaleMale,FemaleFemale},

$\Psi_{2}^{ij}$ is the probability that the age of individuals *i* and *j* belong to an elementary event in the sample space

$$\begin{array}{c} \{ChildChild,ChildAdult,ChildOld,AdultAdult,AdultOld,\\ \;AdultChild,OldOld,OldChild,OldAdult\}, \end{array}$$

and $\Psi_{3}^{ij}$ is the probability determined by the total contact duration between individuals *i* and *j*. In our experiment, we define the susceptibility of individuals getting infected based on their gender and age as follows:

(5) $$\left.\begin{array}{c} P(Male)=2/3,\\ P(Female)=1/3,\\ P(Child)=2/5,\\ P(Adult)=1/5,\\ P(Old)=2/5. \end{array}\right.$$

In that case, for example, P(MaleFemale)=P(FemaleMale)=2/9 and P(AdultAdult)=1/25. In Figure 6 are the results, which are similar to that of Figure 5 in which the weights are dependent on only the contact duration. This probably indicates that the total contact duration between two individuals plays a significant role in determining the weight of the edges.

## 4. Discussions

The study of infectious diseases has been a major research focus in recent years due to COVID-19. Network-based epidemiology is seen as an alternative to equation-based and agent-based models. The project investigates the spread of infectious pathogens using bond percolation theory and high-dimensional network modeling.

Results from equation-based models turn to differ from network-based models where the degree distribution deviates significantly from the mass-action assumption of compartmental models that all susceptible individuals are equally likely to become infected. In that case, the degrees of the nodes are about the same. Equation-based ODE models are generally extremely inexpensive in terms of computational cost. On the other hand, network-based models tend to become expensive as the number of nodes increases.

Network-based models can be easily adapted to incorporate intervention measures such as isolation in comparison to equation-based models. Static contact networks are best used for modeling rapidly spreading pathogens such as Severe Acute Respiratory Syndrome (SARS) and pneumonia. For low-spreading pathogens, adaptive and dynamic contact network models work better [3]. Some work has been done on adaptive networks where the edges or weights vary over time and on temporal networks where the network structure changes with time [16]. The study suggests using the net-based model when a representative network of the population can be determined. It is advisable to store the graph as a linked list rather than an adjacency matrix to reduce the time computational complexity of the method.

## 5. Conclusions

We have used contact network epidemiology to model the transmission of the 1918 bird flu virus for different population data. In the contact network model, the effects of gender, age, and total duration of contacts were combined to determine the weight of the edges. The total duration of contacts seems to have a dominant effect on the weight of the edges compared to gender and age. The contact network model has a more accurate estimation of the overall infected individuals than that of the ODE model. In the future, we hope to determine the values of epidemiological parameters such as infection rate from data using graph neural networks.

## Data Availability

The data and code that support the finding of the study are available on request from the corresponding and the first author.
